# Comparative evaluation of post-mortem methods for detection of diarrhoea in early-life piglets

**DOI:** 10.1186/s40813-026-00523-3

**Published:** 2026-05-26

**Authors:** Cecilie Brandt Becker, Malene Kjelin Morsing, Julie Raundahl Laursen, Emma Amalie Kortemand Hansen, Jens Peter Nielsen, Merete Fredholm, Nicole Bakkegård Goecke, Mette Sif Hansen, Søren Saxmose Nielsen, Henrik Elvang Jensen

**Affiliations:** 1 Section for Parasitology and Pathobiology, Department of Veterinary and Animal Sciences, Faculty of Health and Medical Sciences, University of Copenhagen, Grønnegårdsvej 3, Frederiksberg C, 1870 Denmark; 2 Section for Animal Health and Welfare, Department of Veterinary and Animal Sciences, Faculty of Health and Medical Sciences, University of Copenhagen, Grønnegårdsvej 3, Frederiksberg C, 1870 Denmark; 3 Section for Bacteria and Viruses, Department of Veterinary and Animal Sciences, Faculty of Health and Medical Sciences, University of Copenhagen, Grønnegårdsvej 3, Frederiksberg C, 1870 Denmark

**Keywords:** Perianal faecal staining, Intestinal content morphology, Cotton-swab method, Faecal dry-matter content, Necropsy

## Abstract

**Background:**

Identifying evidence of peri-mortem diarrhoea during necropsy is important for both clinical herd management and research. In live pigs, diarrhoea is typically assessed by inspection of faecal pools on the pen floor scoring of faecal rectal samples, including cotton-swabs, but these methods depend on the availability of faecal material and are therefore often not applicable at necropsy. This study aimed to evaluate intestinal content characteristics and perianal faecal staining as post-mortem indicators of peri-mortem diarrhoea. These indicators were compared with cotton-swab faecal scoring using faecal dry-matter content as a reference. Furthermore, associations with enteric pathogens detected by PCR were investigated.

**Methods and results:**

A total of 145 pigs from a Danish commercial herd were monitored from birth until 10 weeks of age and euthanised in age-stratified batches for post-mortem examinations. Intestinal content from duodenum, jejunum, ileum, caecum, and colon was evaluated for consistency and colour using categorical scales and compared with perianal faecal staining, faecal scores obtained by the cotton-swab method, faecal dry-matter percentage, and pathogen detections in faecal samples by high-throughput real-time PCR. Colonic content consistency and perianal faecal staining were strongly associated, and in all pigs with watery colonic content a perianal faecal staining was observed. Colonic content consistency corresponded well with faecal dry-matter percentage categories, with mucoid, runny and watery consistencies indicating diarrheic cases (sensitivity: 87%, specificity: 55%). Jejunal content consistency showed little to no association with cotton-swab faecal scores, indicating limited value as an indicator of peri-mortem diarrhoea. Pre-weaning piglets predominantly showed lighter (yellow–green), firmer intestinal contents, whereas post-weaning animals displayed darker and looser contents, particularly in the large intestine. Interobserver reliability was high for consistency scoring (Krippendorff’s α > 0.80 across most segments) but low for colour scoring (α < 0.64). Dark colonic content was associated with PCR detection of *Lawsonia intracellularis* and *Brachyspira pilosicoli*, while yellow coloration showed an indirect association with detection of *Clostridium perfringens α* and *β2* toxin genes.

**Conclusions:**

Post-mortem assessment of colonic content consistency represent a promising, practical and reproducible indicator of peri-mortem diarrhoea, but tending to identify a higher prevalence of diarrheic cases compared to diarrhoea defined by dry-matter percentage. Colonic content consistency also showed a strong association with perianal faecal staining. In contrast, jejunal content consistency showed no diagnostic value as an indicator of peri-mortem diarrhoea. Visual assessment of intestinal content colour demonstrated poor interobserver reproducibility, and any observed associations with enteric pathogens should therefore be interpreted with caution. Overall, these findings support the use of colonic content consistency and perianal faecal staining as necropsy-based indicators for detection of peri-mortem diarrhoea.

**Supplementary Information:**

The online version contains supplementary material available at 10.1186/s40813-026-00523-3.

## Background

Diarrhoea remains one of the most prevalent clinical conditions in pig production and presents a significant health and management challenge, particularly during the early life period [1]. Around weaning, many piglets experience post-weaning diarrhoea (PWD), although clinical manifestations of diarrhoea can also be observed in younger and older animals. Diarrhoea is a clinical manifestation defined by the passage of faeces with increased fluidity, typically accompanied by higher volume and increased frequency relative to normal bowel function [2]. In a cross-sectional study of Danish pig production herds, the prevalence of diarrhoea varied substantially, ranging from 10% to 94% [3]. To manage such outbreaks, antibiotic treatment is frequently employed—often at pen, room, or section level rather than individually. While efforts to reduce antimicrobial use in European livestock systems and diminish environmental pollution have led to important regulatory changes, such as the bans on growth promoters [Regulation 1831/2003/EC on additives for use in animal nutrition], and medicinal zinc oxide [4–6], alongside phasing-out of the use of veterinary prescribed colistin [7], further reductions in routine batch treatment may require more targeted interventions.

A shift toward individual treatment strategies depends on an accurate identification of diarrheic animals. This poses a substantial challenge under commercial conditions, where high animal density, intermittent clinical signs, and limited observation time can hinder a reliable diagnosis [8]. Even when direct sampling is attempted, practical and ethical constraints arise. Digital stimulation for faecal collection, though informative, is invasive [3], while cotton swab techniques are less so, but are only validated in the immediate post-weaning period [9]. Moreover, spontaneous faecal samples are not always available, and although some well-established and validated faecal scoring systems exists, there is no universally accepted scoring system for faecal consistency in pigs [10–15], contributing to considerable inter- and intra-observer variability [11, 16].

Other potential diagnostic parameters—such as pH [3, 17], dry matter content [18], colour [10, 12, 17], behaviour [12], and hindquarter soiling [19] —have all been explored, but standardization is not consistently available and the applicability across different age-groups has not been investigated. Additionally, the multifactorial nature of diarrhoea in pigs, involving both microbial, nutritional, and management causes [4, 20–22], further complicates individual-level assessments [23, 24]. Although associations between faecal morphology and specific pathogens have been reported [1, 10], clinical decision-making on farms still largely relies on off-site diagnostics and herd disease history [1].

These diagnostic uncertainties extend into the post-mortem setting, where veterinary pathologists and veterinarians performing on-site necropsies, are often tasked with determining if an animal was suffering from diarrhoea prior to death. Questions about disease presence, severity, and aetiology are central to state the cause of piglet mortality. However, previous studies of Danish piglets have shown inconsistent associations between clinical signs, histopathological findings, and intestinal lesions [25, 26].

The aim of the present study was to investigate whether macroscopic observations at necropsy - including perianal faecal staining and intestinal content characteristics (i.e. consistency) could aid in the identification of piglets that had suffered from diarrhoea perimortem, and whether the colour of intestinal content could be used as an indicator for underlying pathogenic presence. Furthermore, the diagnostic value and observer agreement of these parameters in early life pigs were evaluated, with the goal to improve pathoanatomical diagnoses and support a better herd-level assessment of diarrheic disease dynamics.

## Methods

**Study design and study population:** This study was part of a cohort study approved by The Animal Experiments Inspectorate under the Ministry of Food, Agriculture and Fisheries of Denmark for the project “Intestinal health and the robust pig”, Journal no. 2022-15-0201-01324. The cohort investigated was raised at a commercial, specific pathogen free (SPF) indoor pig production facility in Denmark, which was declared free of pathogens that cause pleuropneumonia, dysentery, atrophic rhinitis, mange, lice, and Porcine reproductive and respiratory syndrome (PRRS). During the cohort period, 15 piglets were randomly selected from each of a total of 170 litters and monitored longitudinally from birth until 10 weeks of age. The piglets were three-way crosses, born to Landrace/Yorkshire sows inseminated with mixed semen from Duroc boars. They were weaned at 26 days of age and subsequently randomized into nursery pens. No cross-fostering was performed, and antibiotic treatments were administered solely on an individual basis, based on clinical indication, with no metaphylactic treatment provided after birth. Due to practical and logistical constraints, observations were carried out across 11 consecutive batches, each comprising between 165 and 270 piglets.

For post-mortem examination and sampling, a subset of 145 piglets was randomly selected using the RAND() function in Excel, resulting in euthanasia of 20, 25, 25, 45, and 30 animals at 4, 14, 25, 46, and 67 days of age, respectively.

**Fig. 1:**
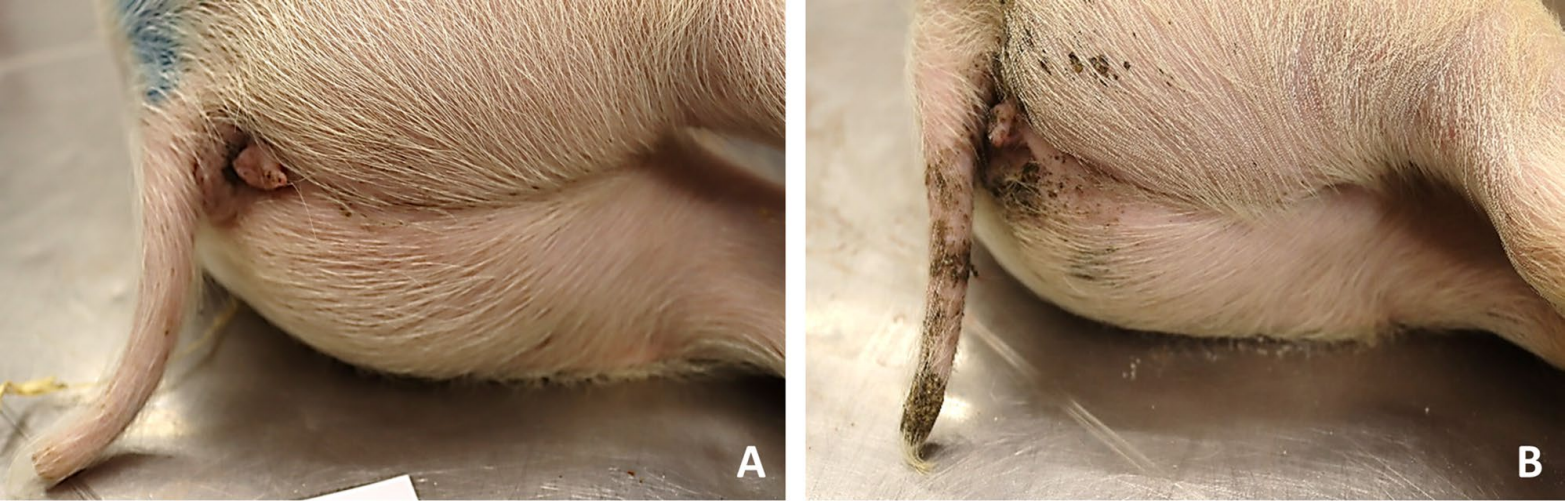
Dichotomous scoring of perianal faecal staining. **(A)** Piglet with no apparent faecal staining of the perianal and tail area, age: 49 days. **(B)** Piglet with noticeable staining of the perianal and tail area, age: 49 days

**Post-mortem assessment and image collection:** Between July 2023 and March 2024, 11 sampling sessions were conducted, each involving 10 to 15 piglets. On the day of sampling, the animals were gently transported in soft bedding for approximately 1.5 h from the herd to the University of Copenhagen, Frederiksberg, Denmark. Upon arrival, the pigs were anesthetized via intramuscular injection of Zoletil (0.1 mL/kg) and subsequently euthanized by intracardiac administration of a pentobarbital overdose. Immediately before euthanasia, faecal consistency was assessed using the “cotton swab method,” in which rectal samples were collected using a cotton swab and scored on a 4-point scale based on the appearance of the material adhering to the swab (Fig. 4, 3rd row) [9]. Carcasses were then examined for visible perianal faecal staining and scored through consensus between three assessors, and photographs of the hindquarters were taken for documentation. Perianal faecal staining was scored as presence or absence of faecal material in the perianal region and/or on the ventral part of the tail (Fig. 1). Following euthanasia, the carcasses were eviscerated, and the intestinal tract was isolated to enable assessment of luminal content from five specific segments: duodenum, jejunum, ileum, cecum, and colon. Anatomical landmarks used for segment identification are illustrated in Supplementary Fig. 1. Each segment was isolated by clamping the oral and aboral ends with haemostats to prevent leakage, then excised and opened longitudinally using scissors and pieces of approximately 2.0 × 2.5 cm were pinned to Styrofoam boards. The content of each segment was photographed for subsequent morphological analysis. Moreover, intestinal content from the descending colon was collected for evaluation of dry matter content. All evaluations were performed within 20 min of euthanasia, and interpretation of individual parameters were blinded to the observers.

**Fig. 2:**
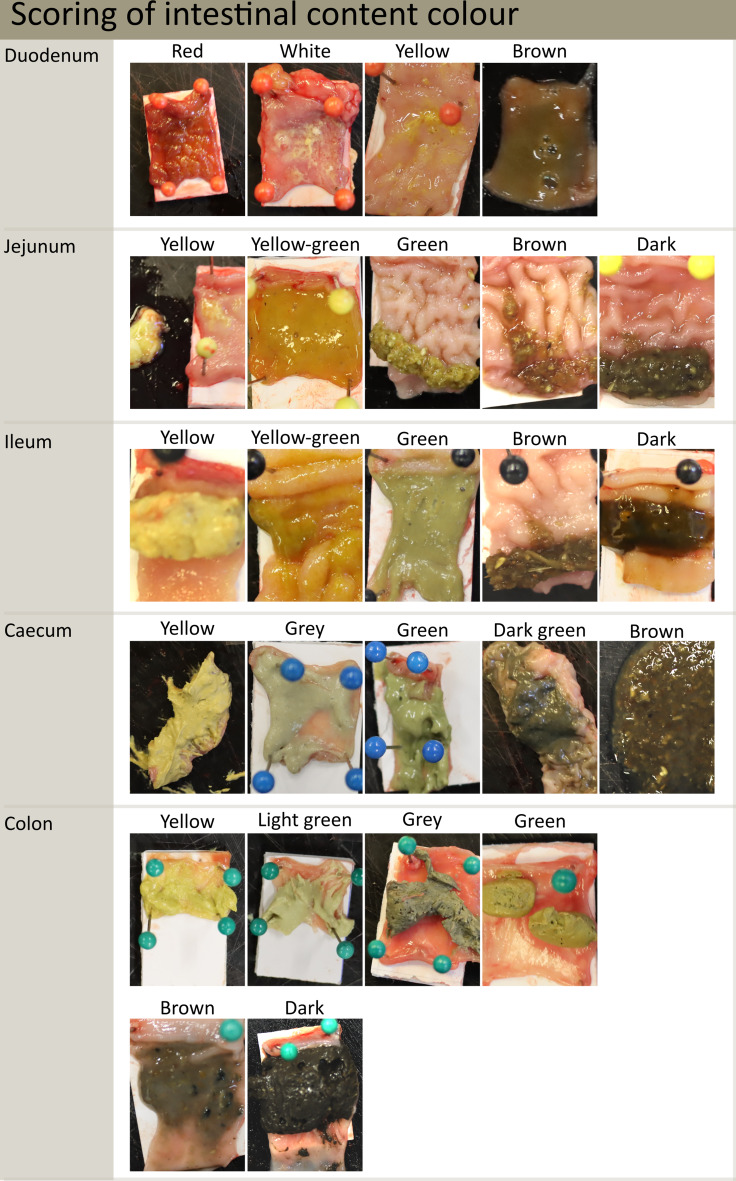
Scoring of intestinal content colour. Score sheet for assessment of intestinal content colour sorted by anatomical location in the gastrointestinal tract

**Fig. 3:**
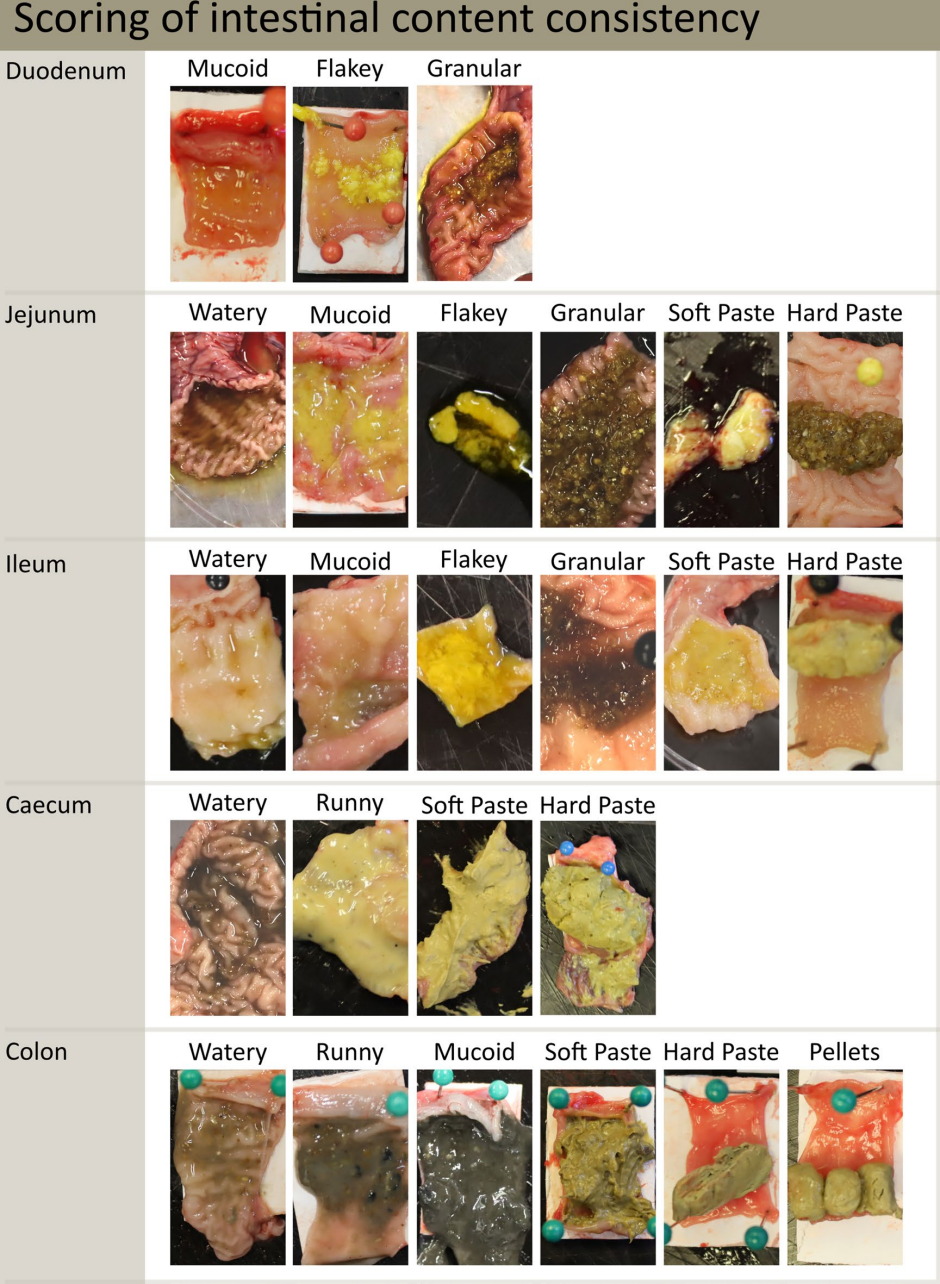
Scoring of intestinal content consistency. Score sheet for assessment of intestinal content consistency sorted by anatomical location in the gastrointestinal tract

**Visual evaluation of intestinal content:** Using the post-mortem images, two categorical scoring systems for intestinal content morphology were developed. The number of categories within each scoring system varied from 3 to 6 categories depending on the anatomical segment assessed. One scoring system rated content consistency, ranging from pellet-like and firm paste to watery appearance, while the second scoring system assessed the colour of the intestinal content. Each intestinal segment was independently scored on both scoring systems by author CBB and scoring was subsequently repeated by authors JRL and EKH to assess interobserver variability. The scoring systems were handed out to the observers prior to evaluation of the images for optimal alignment of categorical terminology. The applied scoring systems for intestinal content morphology can be found in Figs. 2 and 3.

**Fig. 4:**
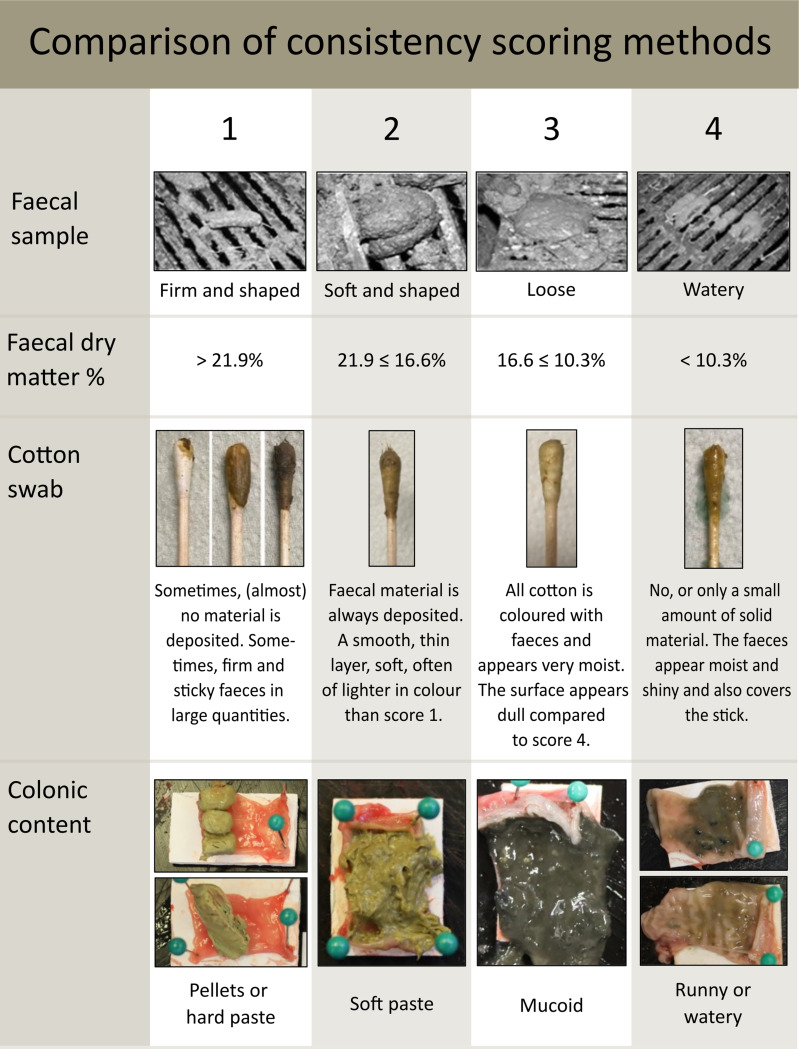
Comparison of four consistency-based diarrhoea assessment methods with translations to a four-point scale. Scores of 1–2 are considered normal, whereas scores of 3–4 are considered diarrhoeic. **First row**: Faecal sample scoring method based on floor samples as published by Pedersen and Toft [11]. Reprinted with permission from Elsevier. **Second row**: Scoring thresholds for faecal dry-matter percentage as suggested by Eriksen et al. [9]. **Third row**: Faecal scoring based on the cotton-swab method described by Eriksen et al. [9]. **Fourth row**: Assessment of diarrhoea based on colonic content consistency. Images of floor-samples and the cotton-swab method are reprinted with permission from copyright holders

**Assessment of diarrhoea using colonic content:** To assess the potential of colonic content as a proxy for faecal material in a necropsy setting, a translation was made between the colonic consistency scores, the faecal scores obtained using the cotton swab method [9], and scoring of faecal samples [11] as illustrated in Fig. 4.

**Quantification of dry matter percentage:** Samples of intestinal content from the descending colon was collected in cryo tubes and stored in the freezer (-80 °C) for subsequent evaluation of dry matter content. Freezing and subsequent thawing of samples were not expected to substantially affect dry matter determination, as the method is based on gravimetric measurement of water loss and does not rely on preservation of structural characteristics, and storage in airtight containers limits water loss due to evaporation. The content from the descending colon was used as a proxy for a passed faecal sample and analysed as previously described by Pedersen et al. [18]. In brief, a plastic container was weighed, and 1 g of thawed intestinal content was added and reweighed. Samples were dried in a microwave oven at low power (120 W) for 30 min, with inspection every 5 min to prevent boiling or burning. The samples were then heated at medium power (385 W) for 10 min, followed by successive 5-min heating intervals until a constant weight was achieved. Samples were weighed between each heating cycle, and dry matter percentage was calculated based on the resulting sample weight loss. The dry matter percentage was converted into faecal score on a 4-point scale using the thresholds suggested by Eriksen et al. [9] presented in Fig. 4.

**High-throughput real-time PCR analysis:** Detection of enteric pathogens and bacterial toxins was performed on a total of 107 rectal swab samples using a high-throughput microfluidic real-time PCR platform (BioMark HD, Standard BioTools, South San Francisco, USA). The complete laboratory protocol, including nucleic acid extraction, primer and probe sequences, along with amplification conditions, is provided in Supplementary Protocol S1 and Supplementary Table 1.

**Statistical analyses:** All statistical analyses were conducted in R [27]. To assess interobserver variability in the scoring of intestinal content, Krippendorff’s alpha coefficient was calculated to evaluate agreement between observers. Intestinal content colour was treated as a nominal variable, while content consistency was evaluated as an ordinal variable. The correlation between faecal score (as assessed by the cotton swab method) and the consistency of jejunal and colonic contents was evaluated using Kendall’s tau, stratified by production stage (suckling pigs and weaners). The correlations between faecal score and intestinal content consistency for the remaining segments were not analysed. To examine the association between colonic content consistency and visible hindquarter soiling, Fisher’s exact test was applied. Due to the presence of zero counts in the contingency tables, P-values were simulated using the simulate.p.value option in R.

Associations between intestinal content colour and the presence of pathogens or bacterial toxins were assessed using Pearson’s χ² test or Fisher’s exact test where appropriate. The pathogens, fimbriae (F) and toxin genes included in the analyses were selected based on segment-specific relevance: *Escherichia coli* F4, *E. coli* F18, and *Clostridium perfringens* α-, β-, and β2-toxins for jejunum; *Lawsonia intracellularis* for ileum; *Brachyspira pilosicoli* for cecum; and *L. intracellularis*,* B. pilosicoli*, and *C. perfringens* α-, β-, and β2-toxins for colon. Rotavirus groups A, B, C, and H were also included. Analyses were performed for grouped colours in each intestinal segment (i.e., Dark [Dark, or Brown], Green [Green, Light Green, or Dark Green], Yellow [Yellow, or Yellow-green]; and additionally Grey for cecum and colon). Analyses were not performed on colour observations from duodenum. For associations identified as significant at P-value < 0.05, relative risk (RR) estimates and corresponding 95% confidence intervals (CIs) were calculated.

Perianal faecal staining was further evaluated as an indicator of faecal score. The measure of caudal contamination [as described above] was modelled as a binary outcome in logistic regression, with faecal score as the explanatory variable, using the glm() function in R. From this model, odds ratio (OR) and 95% CIs were estimated. The sensitivity and specificity of colonic content consistency, cotton-swab scores, and perianal faecal staining as diagnostic indicators of diarrhoea were calculated using faecal dry-matter percentage categories as the reference standard. Because the prevalence of diarrhoea was low in pre-weaning piglets, these measures were only calculated for the post-weaning group.

A P-value < 0.05 was considered statistically significant for all tests. No adjustments were made for multiple comparisons.

## Results

**Intestinal content morphology**: An overview of the assigned scorings for intestinal content colour and consistency is presented in Tables 1 and 2, respectively. In piglets examined prior to weaning, lighter shades—primarily yellow and green nuances—predominated across all intestinal segments. In contrast, post-weaning age groups exhibited darker coloration, particularly within the large intestine. The ileum was frequently found to be empty across all age groups, limiting the ability to assess its content morphology. In terms of consistency, intestinal content was generally firmer in pre-weaning piglets compared to post-weaning animals. Among pre-weaning animals, caecal and colonic contents were most often scored as soft paste or hard paste. At 49 and 67 days of age, however, caecal content was predominantly watery, while colonic content was most frequently categorized as runny or soft paste, respectively.

**Table 1:**
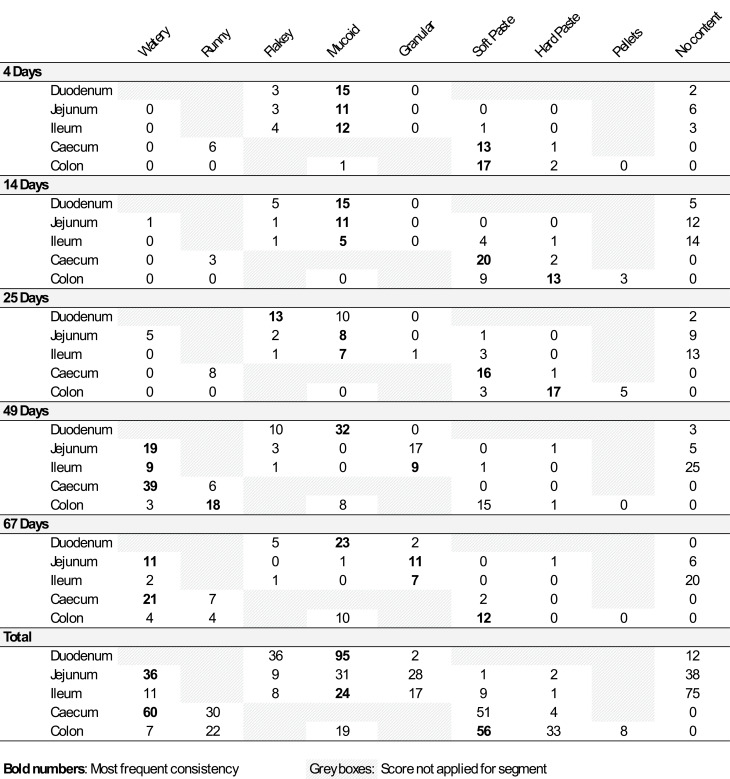
Distribution of intestinal content consistency scores across five intestinal segments and five piglet age groups (*n* = 145)

**Table 2:**
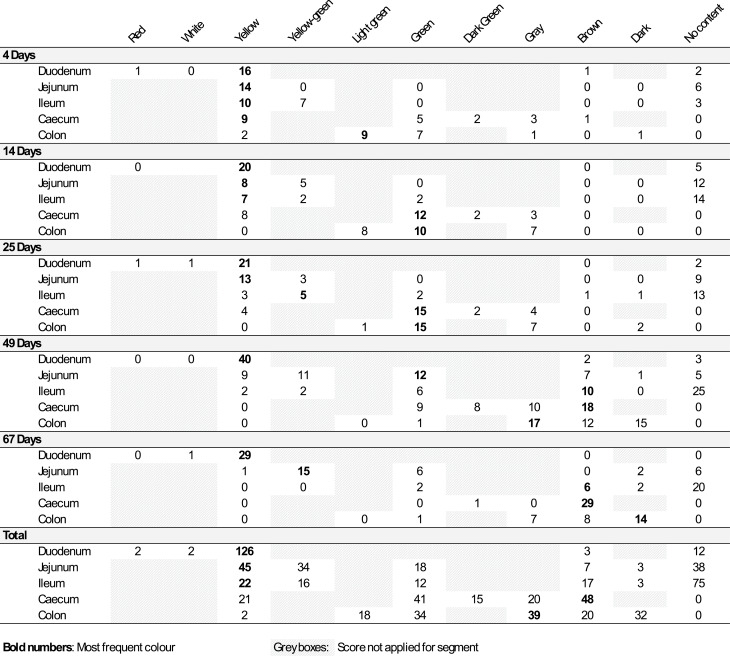
Distribution of intestinal content colour scores across five intestinal segments and five piglet age groups (*n* = 145)

**Interobserver variability**: Krippendorff’s alpha coefficients for observer agreement are summarized in Table 3. For colour scoring, alpha values across segments were below 0.64, indicating poor inter-rater agreement, though still above what would be expected by chance. In contrast, the consistency scoring showed high inter-rater reliability, with alpha values exceeding 0.80 for all segments except the cecum, which showed moderate agreement. Notably, consistency scoring in the colon yielded a Krippendorff’s alpha of 0.815, indicating robust agreement. Furthermore, when specifically evaluating inter-rater agreement for the presence or absence of diarrhoea based on colon content across age groups, all raters concurred in 92% of all cases.

**Table 3:**
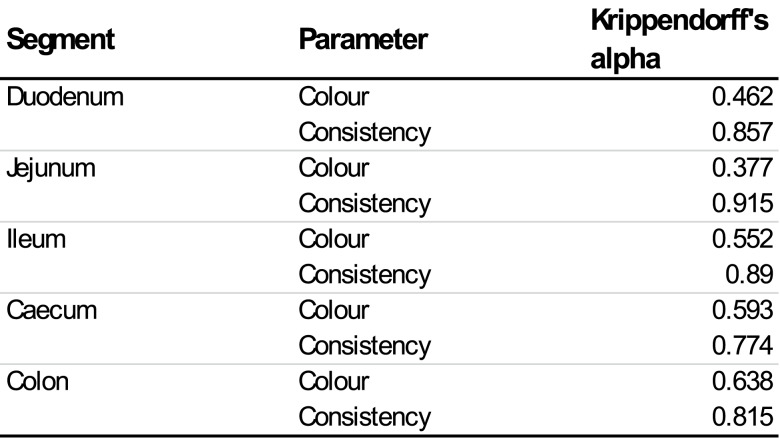
Krippendorff’s alpha coefficients for the inter-rater agreement of intestinal content morphology ratings

**Fig. 5:**
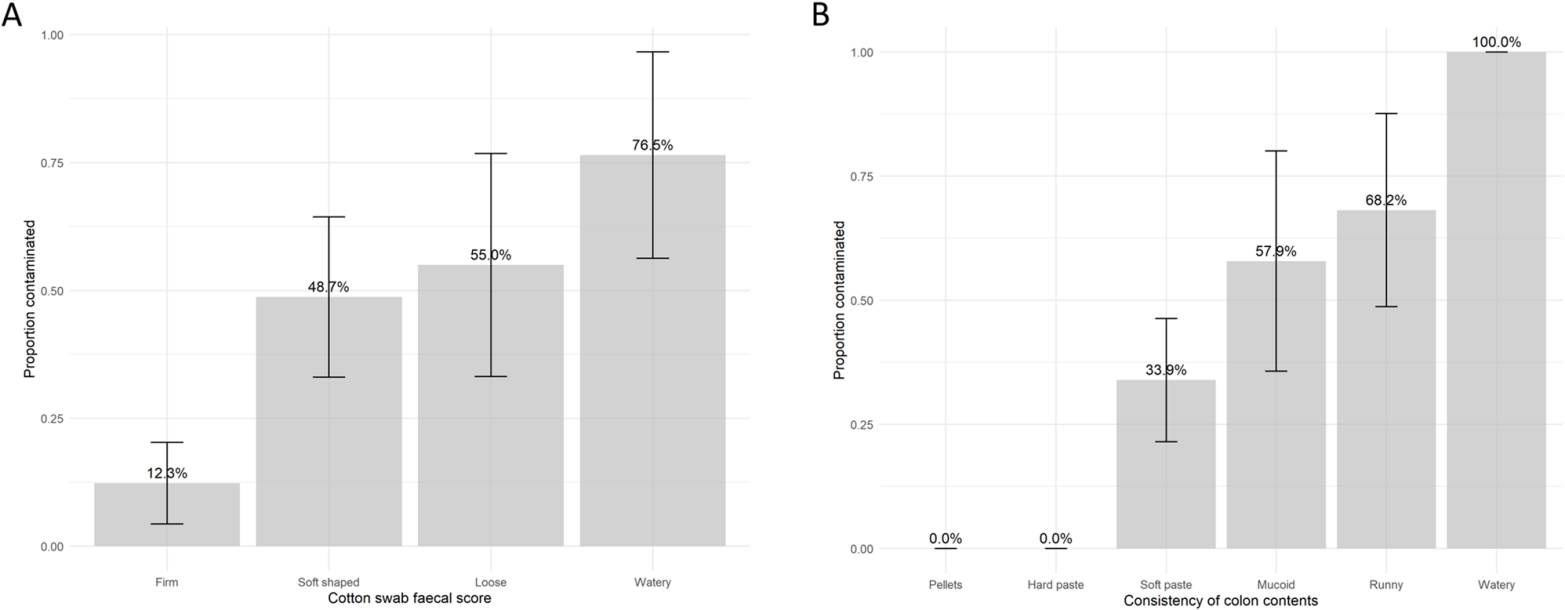
Comparison of perianal faecal staining in piglets across all age-groups sorted by **(A)** the cotton-swab score, and **(B)** the colonic content consistency score. The proportion of stained hind parts is generally increasing with increasing fluidity of the content being evaluated using both methods. Note that the colonic content being categorised as either pellets or hard paste are never associated with faecal staining, while colonic content with a watery consistency is associated with perianal faecal staining in all cases

**Associations between diagnostic techniques**: The overall prevalence of perianal faecal staining in the present study was 36% (52/145), with only five observations of staining prior to weaning. The relationship between perianal faecal staining, cotton swab faecal score, and colonic content consistency revealed a clear and consistent pattern. As shown in Fig. 5, the proportion of animals with visible perianal faecal staining increased as faecal consistency decreased, as assessed by both the cotton swab method and colonic content evaluation. Using the cotton swab method, 76.5% of piglets assigned a score of 4 (indicating watery faeces) exhibited perianal faecal staining, compared to only 12.5% of those with a score of 1 (firm faeces). An even stronger separation was observed with the colonic content consistency score. Animals with colonic contents classified as “Pellets” or “Hard Paste” were never observed with perianal faecal staining, whereas all piglets with “Watery” colonic content exhibited staining. The association between colonic consistency scores and presence or absence of perianal faecal staining was statistically significant (*P* = 0.0005).

When evaluating the correlations between intestinal content consistency (for jejunum and colon) and the faecal score obtained using the cotton swab method, the associations were less pronounced. Cross-tabulations stratified by age group (pre- and post-weaning) can be found in Supplementary Table 2. No overall correlation was observed between jejunal content consistency and the cotton swab faecal score. For colonic content consistency, a moderate overall correlation with faecal score was found (τ = 0.42), primarily driven by post-weaning animals, in which a borderline moderate association was observed (τ=-0.27).

**Comparison of diarrhoea diagnosis**: To evaluate the diagnostic agreement between methods, scoring data were dichotomized into presence or absence of diarrhoea, based on previously defined thresholds, i.e. a faecal score of 1 and 2 were classified as “no diarrhoea,” whereas scores of 3 and 4 were classified as “diarrhoea.” The translation key for colonic content and dry matter percentage in relation to the cotton swab faecal score is illustrated in Fig. 4. For perianal faecal staining, presence of soiling equalled a positive diagnosis. Using faecal dry-matter percentage as the reference standard, the sensitivity and specificity of colonic content, cotton-swab scores, and perianal faecal staining were calculated for the post-weaning group (Table 4).

**Table 4:**
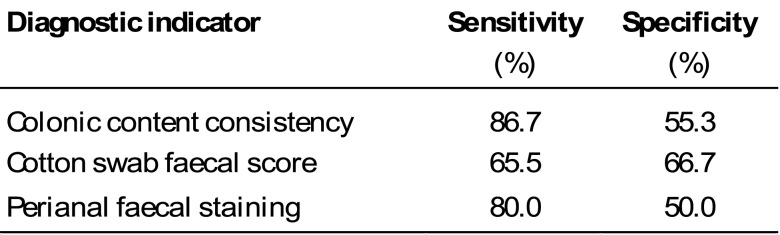
Sensitivity and specificity of diagnostic indicators in reference to dry-matter content diagnosis

The resulting levels of agreement between the four observational parameters are summarized in Table 5 and the individual cross-tabulations can be found in Supplementary Table 3.

**Table 5:**
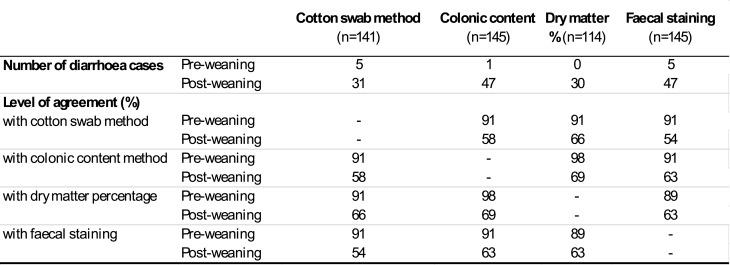
Levels of agreement in the diagnosis of diarrhoea in piglets between four methods

Colonic content and perianal faecal staining were evaluated for all 145 pigs. Four piglets had missing values for cotton-swab scores, and evaluation of dry-matter percentage yielded 31 missing values due to insufficient amount of sampling material. Substantial variation in agreement between methods was observed across the weaning period. Before weaning, all methods consistently indicated a complete or near-complete absence of diarrheic cases. After weaning, however, agreement declined markedly, with perianal faecal staining showing the poorest agreement with the other measures. Agreement between perianal faecal staining and the cotton-swab method was only 54% with slightly higher agreement when compared to colonic content and dry matter percentage. The strongest post-weaning agreement was found between the colonic content and dry matter percentage, which yielded the same diagnosis (diarrhoea or no diarrhoea) in 69% of cases, which is also reflected by a high sensitivity of 86.7%, but a comparatively low specificity of only 55.3%. Although the total number of diarrhoeic cases detected post-weaning was nearly identical for the cotton-swab method and the dry-matter measurements (31 and 30 cases, respectively), the piglets identified were in 33% cases not the same, also reflected in the relatively low sensitivity and specificity.

**Fig. 6:**
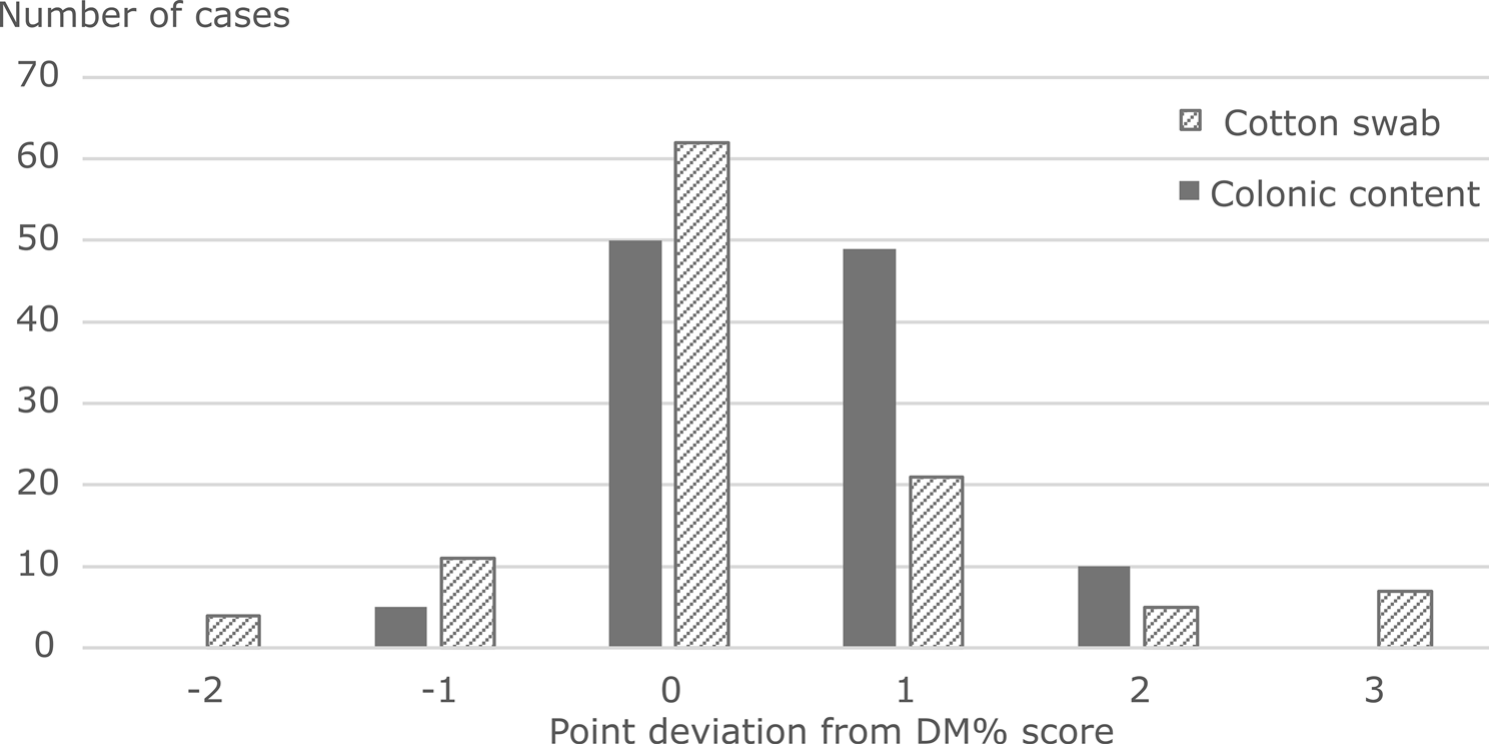
Distribution of discrepancies in assigned faecal scores. Distribution of the discrepancies between faecal scores assigned using the cotton-swab or colonic content method in relation to the score assigned using the dry-matter percentage method. Note that the colonic content tends to assign 1 point higher than the dry-matter content (solid grey bars). The cotton-swab method (striped, grey bars) has a higher number of equal scores but simultaneously shows bidirectional variation of larger magnitude (more − 2, + 2 and + 3)

Evaluation of discrepancies in the assigned faecal scores revealed a consistent pattern between colonic content and dry-matter percentage. When these scores diverged, the colonic score was most often one point higher than the corresponding dry-matter score (solid grey bars in Fig. 6). Samples with a dry-matter score of 2 were frequently assigned a colonic content score of 3, resulting in a higher number of diarrhoeic classifications based on colonic content, explaining the relatively low specificity.

**Fig. 7:**
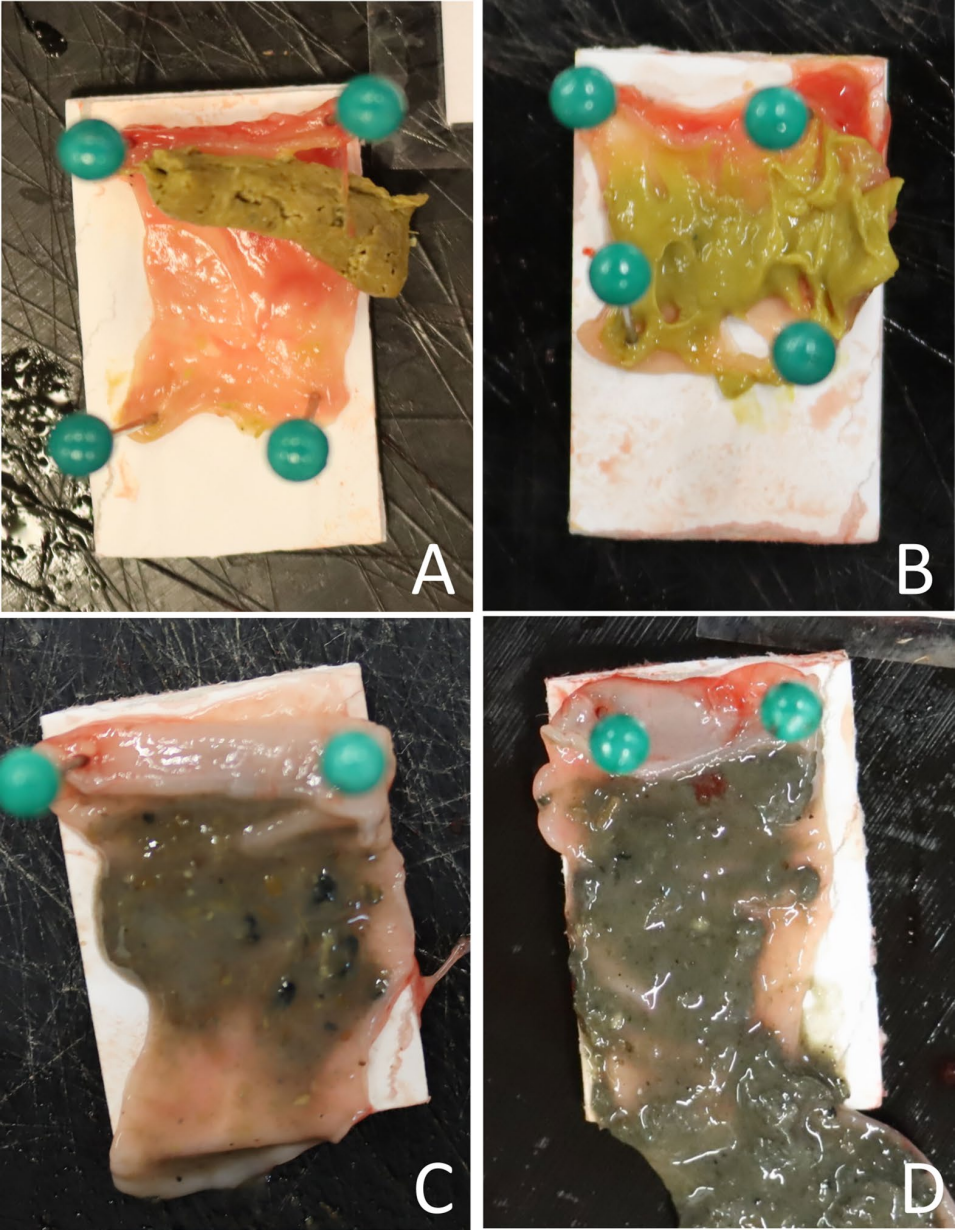
Examples of diarrhoea diagnostic discrepancies. **(A)** Colonic content from a 14 day old piglet, with colonic content score: 1, dry-matter percentage score: 1, and cotton-swab score: 4. **(B)** Colonic content from a 4 day old piglet with colonic content score: 2, dry-matter percentage score: 1, and cotton-swab score: 4. **(C)** Colonic content from a 49 day old piglet with colonic content score: 4, dry-matter percentage score: 4, and cotton-swab score: 2. **(D)** Colonic content from a 49 day old piglet with colonic content score: 3, dry-matter percentage score: 3, and cotton-swab score: 1

Discrepancies between the cotton-swab method and the dry-matter percentage differed in both magnitude and direction (striped, grey bars in Fig. 6). Although a slightly greater number of cases received identical scores compared with the colonic content to dry-matter comparison, deviations between the cotton-swab and dry-matter scores were larger. Seven cases differed by three points on the four-point scale. Deviations occurred in both directions, with cotton-swab scores being either higher or lower than the dry-matter scores. Representative examples of diagnostic discrepancies are provided in Fig. 7.

**Fig. 8:**
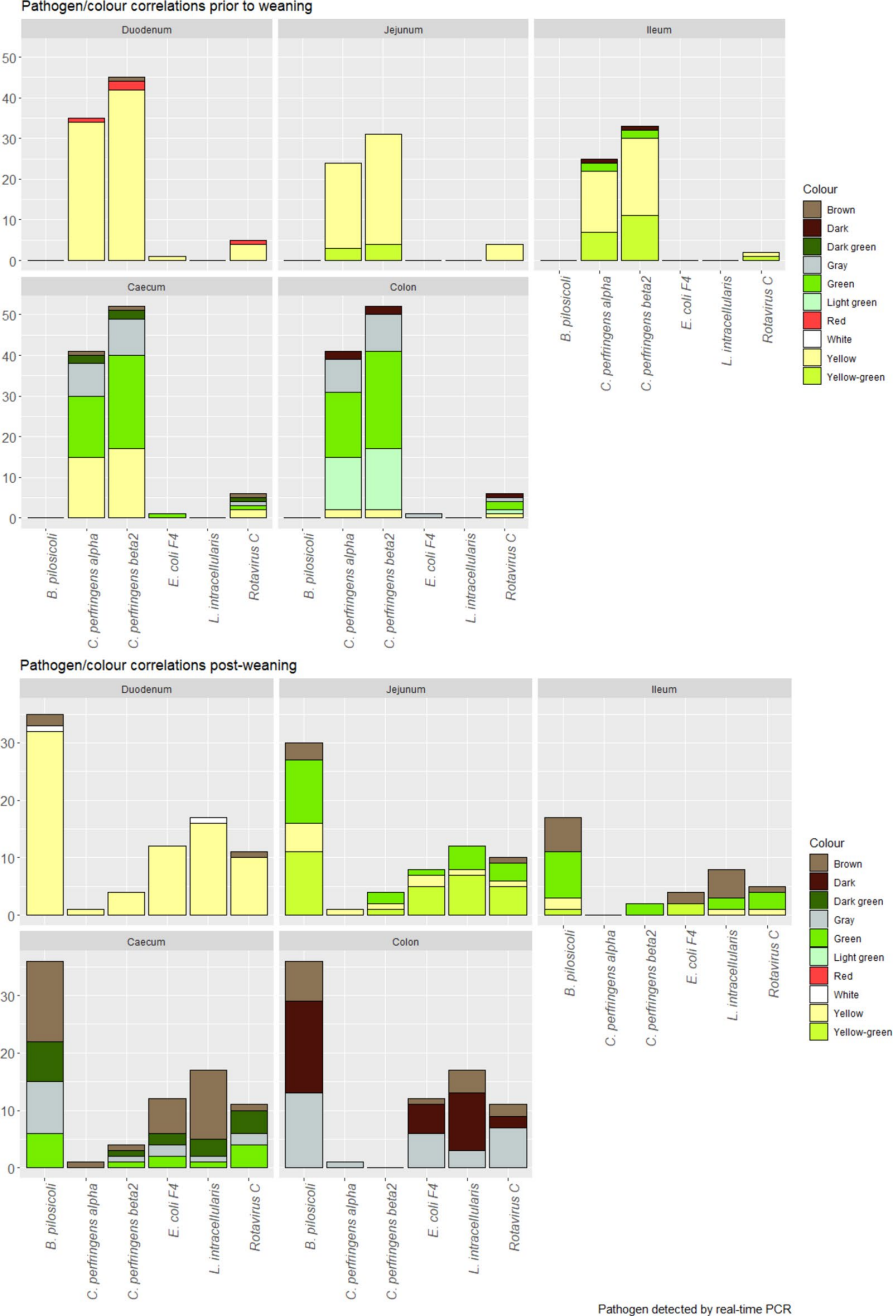
Intestinal content colour profiles across anatomical segments in relation to pathogenic presence pre- and post-weaning. The distribution of intestinal content colours in relation to PCR-positivity is outlined for **Top)** pre-weaning animals and **Bottom)** post-weaning animals

**Association of content colour to diarrhoea and pathogen-status**: Following the high-throughput real-time PCR analysis, no samples were positive for rotavirus B and H, and only two cases were positive for rotavirus A, *E. coli* F18 and *Cl. perf*. β-toxin. Therefore, further analyses were not done for these pathogens. The distribution of observed intestinal content colour in relation to pathogenic presence across the different intestinal segments is outlined in Fig. 8. Dark coloration of ileal, caecal and colonic contents was significantly associated with pathogen detection. In the colon, dark content was linked to a markedly increased risk of testing positive for *B. pilosicoli* (RR = 18.4) and *L. intracellularis* (RR = 22.3). In the caecum, dark coloration was similarly associated with *B. pilosicoli* (RR = 8.2), while in the ileum, dark content was associated with *L. intracellularis* (RR = 6.8). For *C. perfringens* toxins, jejunal colour was strongly linked to yellow, as positive cases were almost exclusively observed in piglets with yellow content (0 cases with dark and only 2 with green content). In the colon, dark coloration was associated with a markedly reduced risk of detecting clostridial toxins (RR = 0.06–0.07), whereas green coloration was associated with a substantially increased risk (RR = 5.7–12.0). The complete 2 × 2 contingency tables with relative risk estimates and p-values are provided in Supplementary Table 4.

**Fig. 9:**
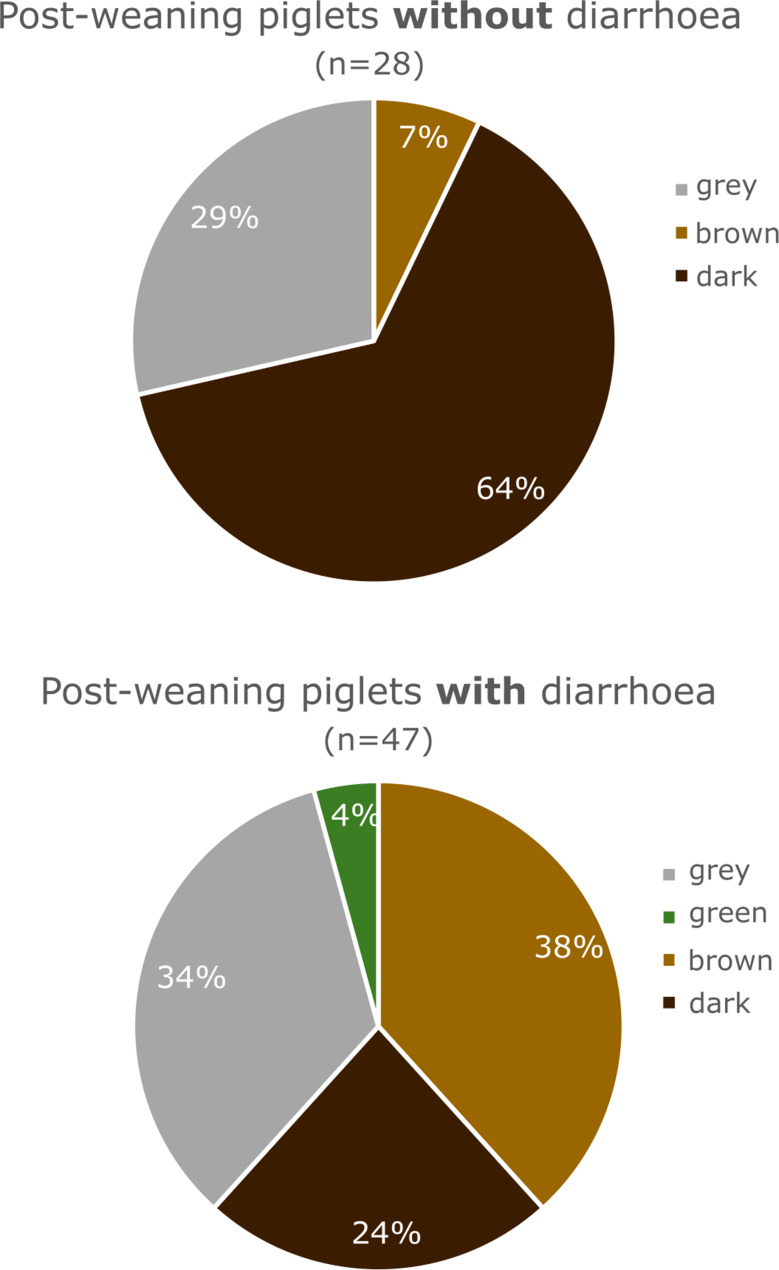
Distribution of colonic content colours in post-weaning piglets. **Top)** The majority of piglets without diarrhoea post-weaning have dark colouration of the colonic content, with fewer showing either grey or brown content. **Bottom)** The distribution of colonic content colours in piglets with diarrhoea is shifted towards lighter shades of brown and grey, and emergence of green colouration as well

When assessing the association between colon content colour and diarrhoea status based on the cotton-swab method in post-weaning pigs, clear differences were observed (Fig. 9). Dark coloration dominated among piglets without diarrhoea (64%), whereas diarrheic piglets had a more even distribution between brown (38%) and grey (34%), with fewer cases of dark coloration (24%). Importantly, green coloration was detected exclusively in diarrheic piglets (4%), in line with findings from a previous study [3].

## Discussion

**Diagnostic value of intestinal content consistency as an indicator of diarrhoea**: Although early-life diarrhoea is commonly linked to small intestinal disease, no meaningful association between jejunal content morphology and faecal scores obtained using the cotton-swab method was found in the present study. This suggests that small intestinal viscosity does not reliably reflect faecal consistency, and therefore, is of limited value as indicator of peri-mortem diarrhoea during necropsies. In contrast, colonic content showed a moderate association with cotton-swab scores and an even stronger association with the dry-matter percentage of content collected from the descending colon. Disagreements between these methods may arise from several factors. Physiologically, the distal colon is the primary site of final water and electrolyte absorption [28], meaning that the segment assessed macroscopically may contain slightly more fluid material than the samples used for dry-matter determination or those obtained by rectal swabbing. Such differences would be expected to result in a unidirectional discrepancy, with the colonic content method tending to assign higher faecal scores. This appeared consistent with our findings: when colonic content and dry-matter scores diverged, the colonic content typically exceeded the dry-matter score by one point. Therefore, colonic consistency may slightly over-estimate the diarrhoea prevalence when compared to dry-matter content categorisation.

However, the discrepancies observed between the cotton-swab and dry-matter methods were larger and bidirectional. In several cases, samples classified as non-diarrhoeic by the colonic content and dry-matter assessments, were assigned a diarrhoeic score based on the cotton-swab method. These inconsistencies could reflect diurnal fluctuations in faecal dry matter [29, 30], intermittent clinical expression of disease, or scoring inaccuracies inherent to the cotton-swab technique. The latter seems plausible, as this method has not been validated for pre-weaning piglets and interpretation can be challenging when only minimal amounts of material is collected on the cotton swab [9]. Furthermore, inter- and intra-observer variability for the cotton-swab method has not yet been characterised, and this could perhaps have contributed to some of the inconsistencies noted in our study. Interestingly, our findings suggest that the inaccuracies may not solely be associated with early age. On the contrary, disagreements in the identification of diarrheic cases were considerably larger post-weaning, compared to disagreements in scores given prior to weaning, perhaps driven by the low prevalence of diarrhoea pre-weaning in this study. The substantial disagreement between diagnostic methods seen in this study, especially post-weaning, highlights the urgency for the development of more reliable tools for the identification of diarrheic animals both post- and ante-mortem. Overall, however, the relatively consistent relationship between visually assessed colonic content and dry-matter percentage suggests that simple macroscopic evaluation can provide an acceptable indication of intestinal content consistency and, by extension, diarrhoeic status.

**Diagnostic value of perianal faecal staining**: The overall prevalence of perianal faecal staining in this study was considerably higher than reported in previous comparable studies [8, 19]. One possible explanation is that earlier research assessed faecal staining directly on-farm, whereas the piglets in this study were transported shortly before examination. Transport-related stress or handling may have caused an inflation of the diarrheic cases observed in this study and secondly contributed to the increased prevalence of perianal faecal staining observed. Despite this, the proportion of soiled piglets was strongly associated with both the faecal scores obtained by the cotton-swab method and the consistency of the colonic content, and – particularly for the latter – clean versus stained hindquarters clearly separated the extremes of firm/pelleted intestinal content from watery content.

Although perianal faecal staining aligned well with the most severe cases, its ability to discriminate between more subtle differences in consistency – particularly between intermediate scores (scores of 2–3) – was limited. Thus, while perianal faecal staining appears to be a helpful indicator for identifying overt watery diarrhoea, it lacks both sensitivity and specificity for detecting mild or early-stage diarrhoeic cases. Our findings therefore support a broader applicability across age groups than previously suggested [19], but primarily for distinguishing severely affected animals from unaffected ones, an applicability that appears to extend into the necropsy room.

**Pathogenic presence predictions by colour of intestinal content**: Colour evaluation is highly subjective due to differences in human colour vision, both in individuals with colour vision deficiencies and within the colour-normal fraction of the population [31]. Even so, several references are linking specific pathogens and microbiological taxa to the colour of faecal or intestinal content [1, 10, 32]. We attempted to link the colour of the intestinal content from five different segments of the intestinal tract to the presence of specific pathogens and bacterial toxins in faecal material using real-time PCR. The scoring of intestinal content colour showed a large interobserver variation with agreements only somewhat above what could be expected by chance, which is in concordance with previous observations of interobserver variability with a larger number of colour options for each category [31, 33]. Therefore, association between pathogenic presence and colour is expected to be highly observer dependent and should be interpreted with care.

In this study, dark coloration of caecal and colonic content was associated with an increased risk of *B. pilosicoli and L. intracellularis*. Yellow coloration of jejunal content was indirectly associated with *C. perfringens*, as toxin-positive samples were almost never observed in piglets with non-yellow jejunal content. In the colon, green coloration was associated with an increased risk of clostridial toxins, whereas dark coloration was associated with a reduced risk. Whether these findings represent true biological relationships remains unclear. Faecal and intestinal content colour is strongly age-dependent, reflecting factors such as dietary shifts from milk to creep feed, mineral supplementation, and microbial community changes [32, 34]. Similarly, pathogens are age-associated, with *C. perfringens*, enterohaemorrhagic *E. coli*, and Rota virus A predominating in neonatal piglets, whereas pathogens such as *B. pilosicoli* and *L. intracellularis* are emerging post-weaning [1]. Thus, the associations observed may be confounded by age. However, marked variation in faecal colour has been documented even within litters under identical environmental and dietary conditions, indicating that additional drivers of faecal coloration exist [17], and disruption of gut homeostasis by pathogens remains a plausible contributor. In summary, these findings indicate that while intestinal content colour may occasionally reflect the presence of pathogens and/or bacterial toxins, it remains a highly subjective parameter and should be interpreted with great caution in applied diagnostics.

**The diagnostic challenge**: Despite the availability of multiple diagnostic approaches, reliable identification of enteric disease in piglets remains challenging in practice. Many commonly applied methods lack sufficient sensitivity to consistently detect affected individuals, but more importantly, often show limited specificity, leading to overestimation of disease prevalence by detection of false positives. This was also reflected in the present study, where all evaluated indicators demonstrated a low specificity when compared to faecal dry-matter measurements. In a clinical setting, such limitations may result in misclassification of healthy animals as diarrhoeic, potentially influencing treatment decisions and contributing to unnecessary or imprecise use of antimicrobials. These findings underline the need for more accurate and validated diagnostic tools that can reliably distinguish between normal physiological variation and clinically relevant diarrhoeic disease in young pigs.

## Conclusion

Assessment of colonic content consistency and perianal faecal staining was found to be promising, practical, and reproducible morphological indicators for identifying peri-mortem diarrhoea in early-life pigs at necropsy. Colonic contents that were watery, runny, or mucoid had a diagnostic sensitivity of 87% compared to diarrhoea based on dry-matter content, and the presence of perianal faecal staining was strongly associated with cases of watery colonic content, although specificity was low for both parameters. In contrast, jejunal content consistency showed little diagnostic value, highlighting that small intestinal content evaluation is not informative for detecting diarrhoea post-mortem. Colonic content and faecal staining aligned reasonably well with faecal dry-matter measurements. Their ability to detect more subtle changes in intestinal consistency was limited, and agreement between diagnostic methods declined post-weaning. The cotton-swab method was also evaluated and showed variable agreement with dry-matter measurements, reflecting the current lack of validation and interobserver reliability data for this diagnostic method. Moreover, visual assessment of intestinal content colour showed poor interobserver reliability, limiting its aetio-diagnostic value in its current form. Taken together, these findings highlight that currently available approaches to diagnose diarrhoea in piglets – both clinical and post-mortem – are constrained by limited specificity, which may lead to overestimation of disease. There is therefore a clear need for the development and validation of more objective, accurate, and reproducible diagnostic tools to support reliable identification of clinically relevant enteric disease in early-life pigs.

## Study limitations

This study was conducted in a single herd, which may limit the generalisability of the findings, as inter-herd variation could not be assessed. Pathogen detection was qualitative, preventing evaluation of whether specific intestinal content characteristics were associated with clinically relevant pathogen loads. This was reflected in the observation that dark colonic content, although associated with *B. pilosicoli* and *L. intracellularis*, was frequently observed in non-diarrheic pigs, making it difficult to distinguish between causal relationships and background colonisation. Observer variation, particularly in colour scoring, also represents a limitation due to the subjective nature of visual assessment. In addition, piglets were transported prior to examination, which may have influenced their diarrhoeic status, although efforts were made to minimise handling stress. Future studies should include multiple herds, incorporate quantitative pathogen data, and explore more objective assessment methods – such as digital image analysis or AI-based classification – to improve the reproducibility and diagnostic value of intestinal content evaluation.

## Supplementary Information

Below is the link to the electronic supplementary material.

Supplementary Material 1

## Data Availability

The datasets used and/or analysed during the current study are available from the corresponding author on reasonable request.

## Abbreviations

OR: Odds Ratio
PCR: Polymerase chain reaction
PRRS: Porcine Reproductive and Respiratory Syndrome
PWD: Post-weaning diarrhoea
RR: Relative risk
SPF: Specific pathogen-free
