# Supplementary material for: Comparative evaluation of post-mortem methods for detection of diarrhoea in early-life piglets
Source: Porcine Health Manag. 2026 May 26;12:42. doi: 10.1186/s40813-026-00523-3 (PMC13421096; doi:10.1186/s40813-026-00523-3)
Supplement: Supplementary file 1 — Supplementary Material 1 [file 40813_2026_523_MOESM1_ESM.docx]

Supplementary material

**6.** **Distal colon**: halfway between the mesenteric artery and rectum.


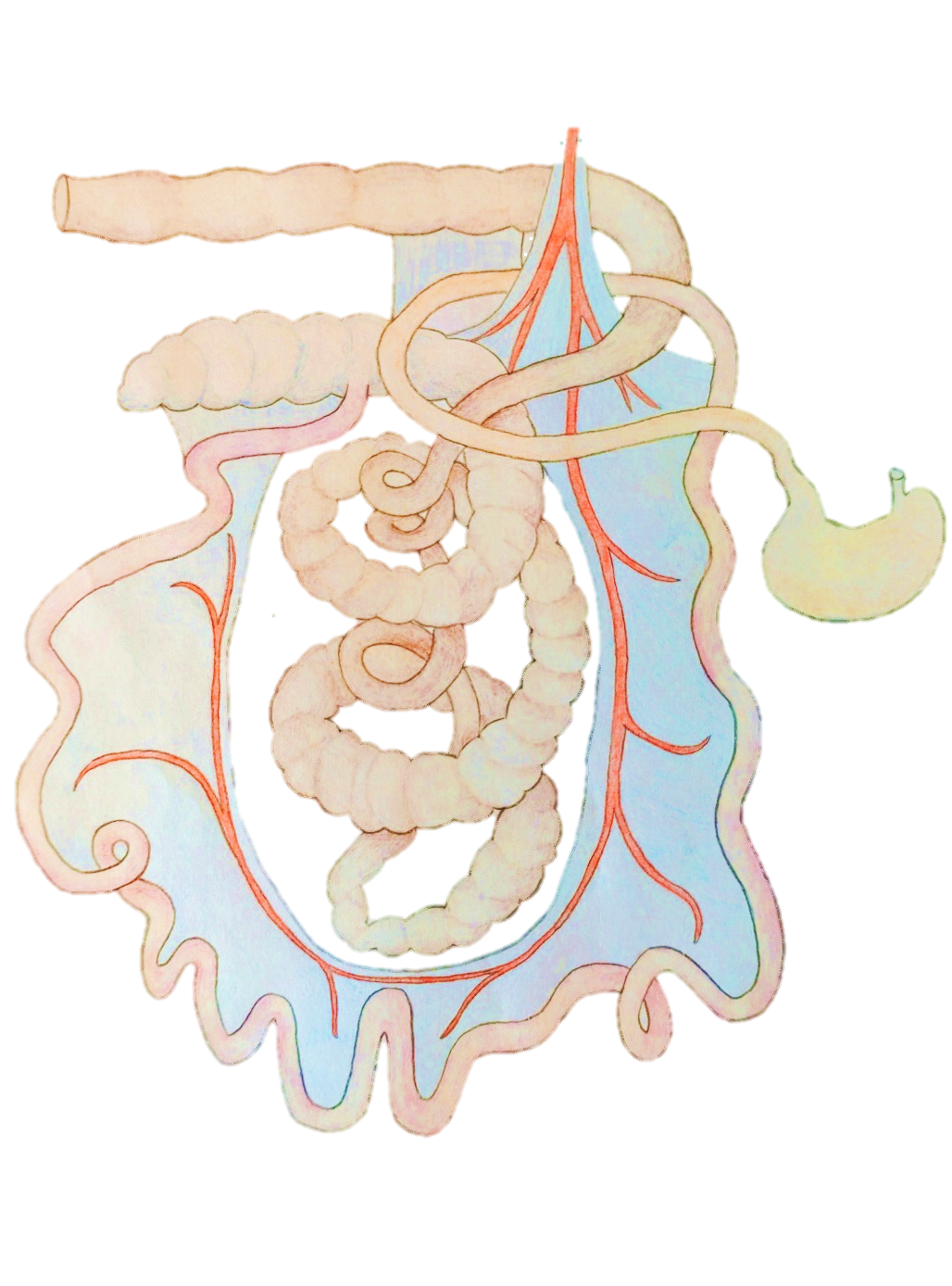


**1. Duodenum**: from the pylorus to the oral aspect of the duodenocolic fold.

**4. Caecum**: mid-caecal area. Halfway between the apex and the ileal inlet.

**3. Ileum**: from the oral aspect of the ileocaecal fold to the ileal inlet.

**2. Jejunum**: approximately halfway between the caudal aspect of the duodenocolic fold and the ileocecal fold.

**5. Colon**: at the central flexure of the colonic spiral.

**Supplementary Figure 1** Sampling sites for evaluation of intestinal content characteristics. Sampling sites 1-5 were used for visual assessment of intestinal content, whereas sampling site 6 was used for evaluation of dry-matter percentage.

**Supplementary protocol S1**

Extraction of nucleic acids: Prior to extraction of nucleic acids, 1.5 mL phosphate-buffered saline (PBS) was added to the tube with the rectal swab. The sample incubated for approximately 5 min at room temperature, followed by vortex for 10 sec and centrifugation at 10,000 x g in 2 min, and 200 μl of the supernatant was subsequently used for extraction. RNA and DNA were extracted from the samples using the extraction robot QIAcube HT (QIAGEN, Hilden, Germany) and the IndiSpin QIAcube HT Pathogen Kit (Indical Bioscience, Leipzig, Germany) using the manufacturer's instructions. Positive and negative (nuclease-free water; Amresco, Cleveland, OH) controls were included in each extraction. The nucleic acids were stored at −80°C until further analysis.

cDNA synthesis and pre-amplification: Prior to high-throughput real-time PCR analysis, the extracted nucleic acids were initially reverse transcribed using a high capacity cDNA RT Kit (Applied Biosystems, Foster city, CA USA). A final volume of 10 µL reaction mix was prepared by mixing 1 µL of 10X RT buffer, 0.4 µL dNTP mix (100 mM), 1 µL of 10X random hexamer, 0.5 µL of MultiScribe RT enzyme, 2.1 µL of nuclease free water and 5 µL of extracted nucleic acid. The cDNA synthesis was carried out in a PCRmax Alpha thermocycler (Cole-Parmer, St Neots, United Kingdom) with the given cycling conditions: 25°C for 10 min, 37°C for 120 min followed by 85°C for 5 min. The cDNA samples were pre-amplified using 2X TaqMan PreAmp master mix (Applied Biosystems). A total volume of 10 µL was prepared by mixing 2.5 µL of cDNA with 5 µL of 2X TaqMan PreAmp master mix (Applied Biosystems) and 2.5 µL of primer mix (200 nM, containing all sets of primers). The pre-amplification was carried out in a PCRmax Alpha thermocycler (Cole-Parmer) using the following program: 95°C for 10 min followed by 14 cycles of 95°C for 15 s and 60°C for 4 min. The pre-amplified products were stored at -20°C until further use.

Pathogen detection by high-throughput real-time PCR: For high-throughput real-time PCR analysis, the BioMark HD (Standard BioTools, South San Francisco, USA) and the 192.24 Dynamic array (DA) integrated fluidic circuit (IFC) chip were used. The 192.24 DA IFC combines 192 samples with 24 assays for 4,608 individual and simultaneous real-time PCR reactions. For information on the 20 PCR assays that were included in this study see **Supplementary Table 1**. A 4 µL sample mix was prepared for each of the samples by mixing 2.2 µL pre-sample mix (prepared by mixing 2 µL of 2X TaqMan Gene Expression Mastermix (Applied Biosystem) and 0.2 µL of 20X sample loading reagent (Standard BioTools)) with 1.8 µL of the pre-amplified sample. Assay mix for each PCR assay was made by mixing 2 µL primer/probe stock (containing 33 µM of each primer and 10 µM of probe) with 2 µL of 2X assay loading reagent (Standard BioTools). Three (3) µL of assay mix and 3 µL of sample mix was loaded into the respective inlets of the 192.24 DA IFC chip. The 192.24 DA IFC chip was placed in the IFC controller RX for loading and mixing for approximately 30 min. Finally, the chip was inserted into the high-throughput real-time PCR platform BioMark HD (Standard BioTools) for thermal cycling with the following cycling condition: 50°C for 2 min, 95°C for 10 min followed by 40 cycles of 95°C for 15 s and 60°C for 60 s. In each chip run, positive and non-template (nuclease-free water) controls were included. Amplification curves and quantification cycle (Cq) values were obtained on the BioMark HD system and finally analysed using Fluidigm Real-Time PCR Analysis software 4.8.1 (Standard BioTools).

**References for supplementary Table 1**

Albini, S., Brodard, I., Jaussi, A., Wollschlaeger, N., 2008. Real-time multiplex PCR assays for reliable detection of Clostridium perfringens toxin genes in animal isolates. Vet Microbiol 127, 179–185. https://doi.org/10.1016/j.vetmic.2007.07.024

Goecke, N.B., Agerlin, M. V, Skadborg, K., Nielsen, E.O., Haugegaard, S., Weber, N.R., Larsen, L.E., 2025. Occurrence and diversity of porcine rotavirus groups A , B , C and H in Danish pigs. Vet. Microbiol. 307, 110615. https://doi.org/10.1016/j.vetmic.2025.110615

Goecke, N.B., Hjulsager, C.K., Krog, J.S., Skovgaard, K., Larsen, L.E., 2020. Development of a high-throughput real-time PCR system for detection of enzootic pathogens in pigs. J. Vet. Diagnostic Investig. 1, 51–64. https://doi.org/10.1177/1040638719890863

Gurjar, A.A., Hegde, N. V, Love, B.C., Jayarao, B.M.Ã., 2008. Real-time multiplex PCR assay for rapid detection and toxintyping of Clostridium perfringens toxin producing strains in feces of dairy cattle. Mol Cell Probes 22, 90–95. https://doi.org/10.1016/j.mcp.2007.08.001

Lindecrona, R.H., Jensen, T.K., Andersen, P.H., Møller, K., 2002. Application of a 5′ nuclease assay for detection of Lawsonia intracellularis in fecal samples from pigs. J. Clin. Microbiol. 40, 984–987. https://doi.org/10.1128/JCM.40.3.984-987.2002

Pang, X.L., Lee, B., Boroumand, N., Leblanc, B., Preiksaitis, J.K., Ip, C.C.Y., 2004. Increased Detection of Rotavirus Using a Real Time Reverse Transcription-Polymerase Chain Reaction (RT-PCR) Assay in Stool Specimens from Children with Diarrhea. J. Med. Virol. 72, 496–501. https://doi.org/10.1002/jmv.20009

Ståhl, M., Kokotovic, B., Hjulsager, C.K., Breum, S., Angen, 2011. The use of quantitative PCR for identification and quantification of Brachyspira pilosicoli, Lawsonia intracellularis and Escherichia coli fimbrial types F4 and F18 in pig feces. Vet. Microbiol. 151, 307–314. https://doi.org/10.1016/j.vetmic.2011.03.013

Starbæk, S.M.R., Rask, M., Brogaard, L., Spinelli, A., Rapson, V., Aagaard, H., Larsen, L.E., Heegaard, P.M.H., Nauwynck, H., Skovgaard, K., 2022. Immunobiology Innate antiviral responses in porcine nasal mucosal explants inoculated with influenza A virus are comparable with responses in respiratory tissues after viral infection. Immunobiology 227, 152192. https://doi.org/10.1016/j.imbio.2022.152192

**Supplementary Table 2** Cross-tabulation of the correlation (Kendall's tau) between the intestinal content consistency in jejunum and colon, and the cotton-swab faecal score i pre- and post-weaning age groups.

**Supplementary Table 3** Cross-tabulations of diagnostic agreement between diarrhoea scoring methods.

**Supplementary Table 4** Cross tabulations of pathogen-colour correlations sorted by anatomical segment.
